# Hypoxia Precondition Promotes Adipose-Derived Mesenchymal Stem Cells Based Repair of Diabetic Erectile Dysfunction via Augmenting Angiogenesis and Neuroprotection

**DOI:** 10.1371/journal.pone.0118951

**Published:** 2015-03-19

**Authors:** XiYou Wang, CuiLong Liu, ShaoDan Li, Yong Xu, Ping Chen, Yi Liu, Qiang Ding, Wasilijiang Wahafu, BaoFa Hong, MingHui Yang

**Affiliations:** 1 Department of Traditional Chinese Medicine, Chinese People’s Liberation Army General Hospital, No 28 Fuxing Road, Hai dian District, Beijing 100853, People’s Republic of China; 2 Department of Urology, PLA Navy General Hospital, No 6 Fucheng Road, Hai dian District, Beijing 100048, People’s Republic of China; 3 Department of Urology, Chinese People’s Liberation Army General Hospital, No 28 Fuxing Road, Hai dian District, Beijing 100853, People’s Republic of China; Center for Interdisciplinary Research in Biology (CIRB) is a novel Collège de France / CNRS / INSERM, FRANCE

## Abstract

The aim of the present study was to examine whether hypoxia preconditioning could improve therapeutic effects of adipose derived mesenchymal stem cells (AMSCs) for diabetes induced erectile dysfunction (DED). AMSCs were pretreated with normoxia (20% O_2_, N-AMSCs) or sub-lethal hypoxia (1% O_2_, H-AMSCs). The hypoxia exposure up-regulated the expression of several angiogenesis and neuroprotection related cytokines in AMSCs, including vascular endothelial growth factor (VEGF) and its receptor FIK-1, angiotensin (Ang-1), basic fibroblast growth factor (bFGF), brain-derived neurotrophic factor (BDNF), glial cell-derived neurotrophic factor (GDNF), stromal derived factor-1 (SDF-1) and its CXC chemokine receptor 4 (CXCR4). DED rats were induced via intraperitoneal injection of streptozotocin (60 mg/kg) and were randomly divided into three groups—Saline group: intracavernous injection with phosphate buffer saline; N-AMSCs group: N-AMSCs injection; H-AMSCs group: H-AMSCs injection. Ten rats without any treatment were used as normal control. Four weeks after injection, the mean arterial pressure (MAP) and intracavernosal pressure (ICP) were measured. The contents of endothelial, smooth muscle, dorsal nerve in cavernoursal tissue were assessed. Compared with N-AMSCs and saline, intracavernosum injection of H-AMSCs significantly raised ICP and ICP/MAP (p<0.05). Immunofluorescent staining analysis demonstrated that improved erectile function by MSCs was significantly associated with increased expression of endothelial markers (CD31 and vWF) (p<0.01) and smooth muscle markers (α-SMA) (p<0.01). Meanwhile, the expression of nNOS was also significantly higher in rats receiving H-AMSCs injection than those receiving N-AMSCs or saline injection. The results suggested that hypoxic preconditioning of MSCs was an effective approach to enhance their therapeutic effect for DED, which may be due to their augmented angiogenesis and neuroprotection.

## Introduction

Erectile dysfunction (ED), often referred as impotency in men, is defined as the inability to acquire and/or sustain a sufficient erection function to achieve satisfactory sexual intercourse. One of major risks for ED is diabetes mellitus (DM). It has been demonstrated that diabetic male patients suffered a higher ratio of ED compared with nondiabetic men [1,2,3,4]. The causes of ED in diabetic men were complicated and were commonly attributed to functional impairments in blood vessel, muscle, and nerve [5]. DM often induced oxidative stress damage in cavernosum tissues, which may cause the endothelium to loss its physiological properties and shift a vasoconstrictor, prothrombotic and pro-inflammatory state [6,7]. The dysfunction of vascular endothelium is thus considered to play a major role in the early development of diabetic erectile dysfunction (DED). Moreover, smooth muscle dysfunction is observed when DED happens, which may contribute to oxidation of low density lipoprotein (LDL) and increased production of reactive oxygen species (ROS) [8].

In the past years, various strategies have been proposed for treating DED. The predominant treatment options are oral medications and phosphodiesterase type 5 inhibitors, such as Tadalafil, Vardenafil, Sildenafil [9]. However, only about half of DED patients embrace improvements from these drugs. More seriously, common side effects of these drugs, including headache, flushing, dyspepsia, nasal congestion, abnormal vision and diarrhea, also need to be tolerated by patients following treatments [5]. Other non-drug based strategies, such as vacuum constriction devices and penile prosthesis implantation, have emerged, but limited advantages were achieved due to the inability to refresh functional cells and tissues [5,10]. Therefore, there is a large interest in developing a novel therapy technique to recover normal functions of damaged endothelial cells (ECs) and cavernous smooth muscle cells (SMCs).

Alternatively, stem cell-based therapy is considered to be one of the promising strategies to enhance tissue repair and functional recovery for patients with DED. Among candidate stem cells for transplantation in DED patients, mesenchymal stem cells (MSCs) are believed to possess large potential, owing to their abundant autologous availability, thus avoiding ethical conflicts and graft rejection [11]. Importantly, investigations showed that successful transplantation could also be achieved from allogeneic MSCs [12], indicating a low immunogenicity of the cells [13]. Moreover, transplantation of MSCs provides many other benefits. One of typical advantages is that they can secrete a variety of cytokines which exert general protective effects *in vivo* [12,13,14,15], including glial cell line-derived neurotrophic factor (GDNF), brain-derived neurotrophic factor (BDNF),nerve growth factor (NGF) and vascular endothelial growth factor(VEGF) [16,17]. As for ED therapy, many successful efforts have been made in the area of MSCs implantation. In animal studies, convincing evidence shows that transplantation of MSCs resulted in a beneficial effects on reversing age-related ED [18], such as enhancing functional recovery by inhibiting apoptosis [19]. In terms of DED, MSCs based treatment also exhibited encouraging effects [20]. Furthermore, it has been revealed that intracavernous transplanted MSCs restored erectile function of diabetic rats mainly through increasing endothelium and smooth muscle content in the corpus cavernosum [21,22].

Recently, various strategies have sprung up to enhance the therapeutic potentials of MSCs. Among those strategies, hypoxic preconditioning (HP) was demonstrated to comprehensively increase the expression of endogenous defense/regenerative genes in stem cell therapy [23,24,25,26]. Previous studies have successfully applied the strategy in stem cell therapy of several diseases, such as hypoxic preconditioning MSCs for myocardial infarction. More importantly, significant benefits on stem cell therapies by hypoxic preconditioning were observed by independent groups [23,24,25,26]. However, whether hypoxic preconditioning could enhance MSC based treatment of ED has not been demonstrated.

In the present study, the hypothesis is proposed that the hypoxic preconditioning can efficiently improve the therapeutic potentials of MSCs for DED through augmenting their paracrine effects. To test our hypothesis, the study was designed and the efficacy of normal MSCs and HP preconditioned MSCs were systematically compared for treatment of DED.

## Materials and Methods

### Ethical Approval

All animals were raised and handled according to the National Institutes of Health Guidelines on the Use of Laboratory Animals. The animal experimental protocol was approved by the Committee on the Ethics of Animal Care and Use of Chinese PLA general hospital. All surgeries were performed under anesthesia, and all efforts were made to minimize suffering. Rats were anaesthetized by intraperitoneal injection of pentobarbital sodium (30 mg/kg)

### Isolation and cultivation of adipose derived stem cells

Isolation and culture of adipose derived stem cells were conducted as previously described [27,28]. Briefly, male Sprague-Dawley rats (80–120g) were killed; adipose tissues were obtained from inguen and washed with PBS to remove bloodiness. The tissues were cut into 1mm×1mm size and digested with collagenase I. After centrifugation, the resulted cells were suspended in DMEM containing 10% fetal bovine serum/ 1% penicillin-streptomycin/2 mM L-glutamine, and incubated in humidified atmosphere and 5% CO_2_ at 37°C for 48h. The medium containing non-adherent cells were then removed and fresh culture medium was added. The medium was changed every 3 days. Cells were passaged when they reached about 90% confluence and were used at passage 3. To confirm the cellular identity of cultured cells, fluorescence-activated cell sorting was performed using CD90, CD29, CD34 and CD45 markers.

### Hypoxia protocol and normoxia control

Hypoxic preconditioning of cells was achieved with a well characterized, finely controlled ProOx-C-chamber system (Biospherix, Redfield, NY) for 24 h. The O_2_ concentration in the chamber was controlled by the ProOx model 110 and maintained at 1%. Cells of normoxia treatment were in consistent with the same procedures except their exposures to the 20% O_2_ during the whole preparation.

### Multipotent Differentiation

To determine the multipotent differentiation capacity of AMSCs, the experiments of adipogenic and osteogenic differentiation were conducted with induction medium for 3 weeks as following:

**Adipogenic induction**. AMSCs were seeded at 20,000 cells/cm^2^ and inducted with alpha MEM medium containing 10% FBS, 1% penicillin and streptomycin, 1mM dexamethasone, 500mM 3-isobutyl-1-methylxanthine, 10 mg/ml insulin, and 100mM indomethacin for 21 days. Differentiated cells were then fixed with 4% paraformaldehyde for 30 min at room temperature and stained with fresh Oil Red O solution for 50 min. A light microscope was applied to identify fat droplets.

**Osteogenic induction**. AMSCs were seeded at 5,000 cells/cm^2^ and inducted with alpha MEM medium with 100 nM dexamethasone, 10 mM β-glycerophosphate, and 50 mM ascorbic acid-2-phosphate (Wako Chemicals, Richmond, VA) containing 10% FBS and 1% penicillin and streptomycin for 21 days. Differentiated osteocytes were then fixed by ice-cold 95% ethanol for 5 minutes at room temperature and stained for calcium deposits with 2% Alizarin Red Solution (pH = 4.0). Light microscopy was utilized to recognize orange-red stained areas that suggested calcium deposits.

### Establishment of type I diabetic rats

After an overnight fast, 55 Sprague-Dawley rats (10 weeks old) received intraperitoneal injection of a citrate acid buffer solution (pH = 4.0) containing streptozotocin (STZ; 60 mg/kg; Sigma, St Louis, Missouri) to induce type I diabetes according to the previous protocol [29]. The remaining 15 rats were injected with an equivalent volume of citrate buffer solution and acted as nondiabetic controls. Blood samples were collected from tail prick for blood glucose measurement after 72 hours. Those rats with a blood glucose level higher than 200 mg/dL were selected as diabetic rats.

### Intracavernous administration of MSCs

Eight weeks after STZ injection, Apomorphine (APO, 100 ug/kg) (Sigma) was employed to screen the ED rats according to Heaton’s method [30]. After the APO subcutaneous injection, 47/55 (85.45%) rats were finally determined as diabetic ED rats. The rats were then randomly divided into three experimental groups: the saline group received left corpus cavernosum (CC) injection of 300 μL cell-free PBS solution; the normoxia group received CC administration of 300 μL PBS solution containing 1×10^6^ normoxia AMSCs; the hypoxia group received CC administration of 300 μL PBS solution containing 1×10^6^ hypoxia AMSCs. All implantation operations were performed under the same anesthetic procedure. Animal numbers were 15 to 16 in each group. Prior to aseptic transplantation, all rats were anesthetized with ketamine (30 mg/kg) and xylazine (4 mg/kg).

### Erectile function assessment

Erectile function was determined by mean arterial pressure (MAP) and intracavernosal pressure (ICP) at the 4th week as previously described [31]. Briefly, rats were firstly anesthetized by ketamine (30 mg/kg) and xylazine (4 mg/kg). A PE-50 tube filled with 250 IU/ml heparinized saline was then cannulated into their exposed left carotid artery and connected to a pressure channel to continuously monitor MAP. For the measurement of ICP, firstly, both the penile crus and cavernous nerve (CN) were exposed through a lower midline abdominal incision. A heparinized (heparin 250 IU/ml) 25-G needle connected to another pressure transducer for physiological recording was then inserted into left crura. The stimulation around CN was performed through a bipolar electrode controlled by an electrical stimulator (ShangHai Biowill Co., Ltd. Shanghai, China), which was utilized to produce monophasic rectangular pulses (a fixed width of 0.2 ms, 1.5 mA, frequency at 20 Hz, and duration of 50 s). Every ten minutes, electrostimulations were repeated for three times and continuous simultaneous recordings of MAP and ICP were performed and recorded using a PC Lab (ShangHai Biowill Co., Ltd. Shanghai, China) signal process system. The highest ICP was chosen for statistical analysis. The ratio of peak ICP/MAP was calculated to express the erectile responses.

### Real time PCR analysis

For cultured AMSCs cells, total RNA isolated from at least 1×10^5^cells by using RNeasy minikit (Qiagen, Hilden, Germany) according to manufacturer’s protocol. After quantification, RNA samples with A260 /A280 nm ratio more than 1.8 were retained and applied in the following experiments. 2 μg of total RNA was reversely transcribed into 1^st^-strand cDNA with PrimeScript RT reagent kit (Takara, Shiga, Japan). The obtained cDNA was then subjected to RT-PCR analysis and the sequence of primers for PCR was as follows: GAPDH, 5’-ACC ACA GTC CAT GCC ATC AC-3’ (sense) and 5’-TCC ACC ACC CTG TTG CTG TA-3’ (anti-sense); HIF-1α, 5’-AAG TCT AGG GAT GCA GCA C-3’ (sense) and 5’-CAA GAT CAC CAG CAT CTA G-3’ (anti-sense); VEGF, 5’-GTG GAC ATC TTC CAG GAG TA-3’ (sense) and 5’-TCT GCA TTC ACA TCT GCT GT-3’ (anti-sense); Ang-1, 5’-CAC CGT GAG GAT GGA AGC CTA-3’ (sense) and 5’-TTC CCA AGC CAA TAT TCA CCA GA-3’ (anti-sense); Flk-1, 5’-AAT GCC CAT GAC CAA GAA TGT-3’ (sense) and 5’-GGA TAG AGC CGC GTG TCT GAA-3’ (anti-sense); bFGF, 5’-AGA GCG ACC CAC ACG TCA AAC TAC A-3’ (sense) and 5’-ATG GCC TTC TGT CCA GGT CCC G-3’ (anti-sense); BNDF, 5’-CCA TAA GGA CGC GGA CTT G-3’ (sense) and 5’-GAC ATG TTT GCG GCA TCC A-3’ (anti-sense); GDNF, 5’-TAT CCT GAC CAG TTT GAT GA-3’ (sense) and 5’-TCT AAA AAC GAC AGG TCG TC-3’ (anti-sense); CXCR4, 5’-AAA GCT AGC CGT GAT CCT CA-3’ (sense) and 5’-CAC CAT TTC AGG CTT TGG TT-3’ (anti-sense); SDF-1, 5’-AAA CCA GTC AGC CTG AGC TAC-3’ (sense) and 5’-TTA CTT GTT TAA AGC TTT CTC-3’ (anti-sense). In addition, PCR products were analyzed with agarose gel electrophoresis. The relative level of gene expression in AMSCs was quantified and compared between normoxia AMSCs and hypoxia ones.

### Western blotting

AMSCs treated with or without hypoxia were lysed in Laemmli Sample Buffer (Bio-Rad). Proteins were collected by centrifugation and concentrations were determined by BCA Protein Assay Kit (Thermo Scientific). Proteins were loaded on sodium dodecyl sulfate polyacrylamide gel for electrophoresis. After proteins were transferred to nitrocellulose membranes; primary antibodies against VEGF (abcam), bFGF (abcam), HIF-1α (Cell signaling technology) or BNDF (abcam) were incubated over night at 4°C. Then, corresponding secondary antibodies were incubated for 1 h at room temperature. GAPDH was used as internal standard.

### Histology and Immunohistological analysis

The middle parts of the rat penile shafts (2.5 mm) were obtained and fixed in 4% paraformaldehyde, dehydrated by an ethanol gradient, and then embedded in paraffin. 5μm paraffin-embedded sections were prepared. Masson’s trichrome staining was performed to assess the collagen deposition (tissue scarring). For immune staining with CD3 (abcam), anti-CD31 (Sigma), vWF (Sigma), αSMA (Sigma) and nNOS antibodies, images of 100× magnification was taken (Santa Cruz Biotechnology, Inc.). Quantitative image analysis was conducted through computerized densitometry utilizing the ImagePro program (version 6.3) coupled to a Leica microscope. Using the software, the vessel perimeters and the positive stained area around the vessels could be quantified. The unit of average vessels perimeter calculated by ImagePro software was μm. The expression levels of vascular markers (CD31, vWF and α-SMA) around vessels were expressed as positive stained area/vessel perimeter. Then, the expression levels of vascular markers in each were standardized as the ration to normal. Eight sections were counted for every rat penile and five fields were taken from each section.

### Statistical analysis

Values were expressed as mean±standard deviation (SD). *P* values<0.05 were considered as statistically significant. Comparisons of weight, blood glucose, ICP, ICP/MAP, and immunofluorescence staining analysis between the groups were performed by using 1-way ANOVAs followed by Tukey’s post hoc test for multiple pair-wise examinations.

## Results

### Characteristics of AMSCs

**Fig 1:**
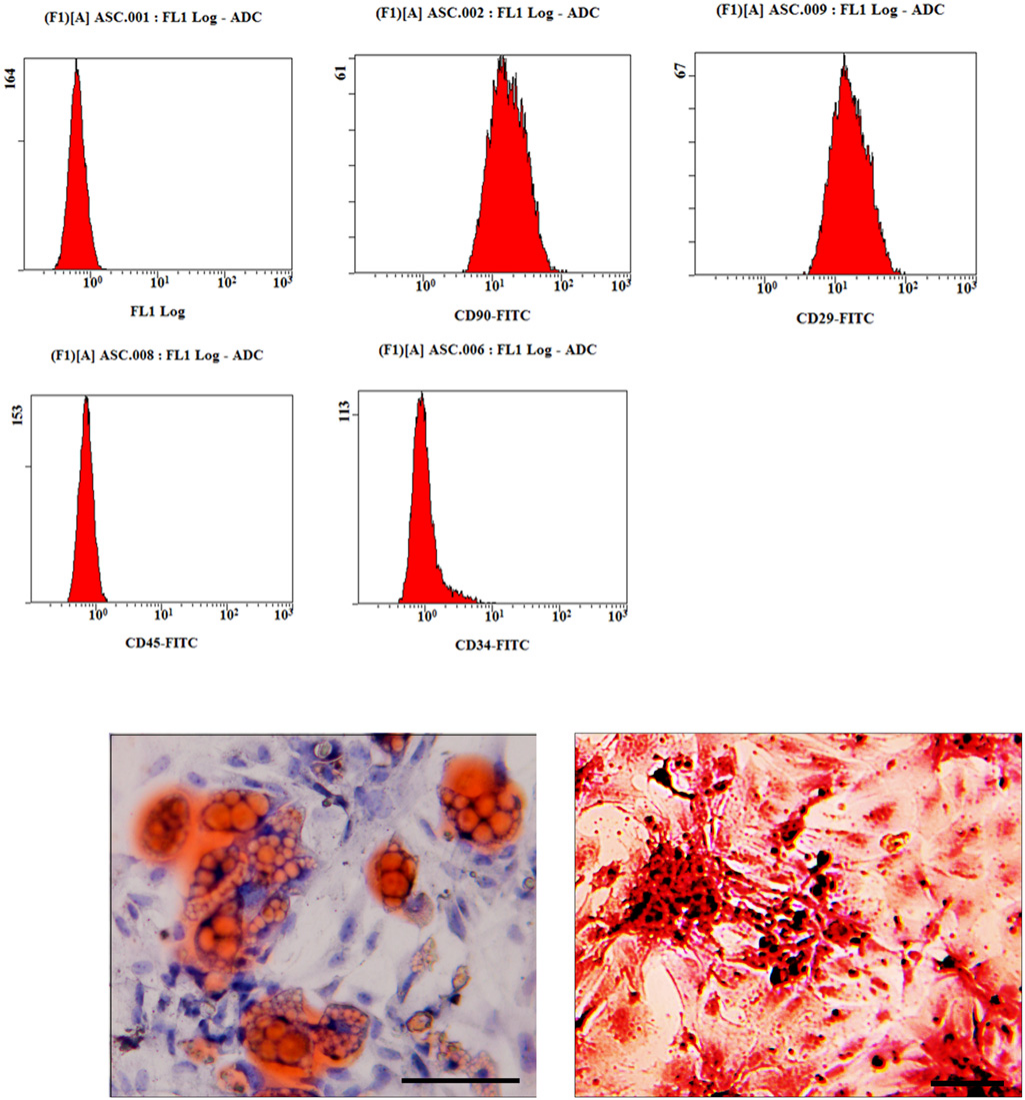
Characterizatio of rat AMSCs. A, flowcytometery demonstrated that most AMSCs express CD90, CD29 and do not express CD45, CD34; Furthermore, no significant difference was found between AMSCs and hypoxia-treated AMSCs; B, After culture in differentiating medium, AMSCs differentiated in to adpogenic and osteogenic lineages and differentiated cells could be positively stained by Oil red O staining and Alizarin red S staining (Scale bar: 50μm).

AMSCs were isolated from ten-week old rats. Using the previous established method, we systematically characterized the immune phenotype and differentiation potential of AMSCs in our investigations [21]. Typically, Cells in our study were selected at passage 3. The surface markers of these cells were identified by fluorescence-activated cell sorting (FACS). Most AMSCs expressed CD90, CD29, but not CD45 and CD34, (Fig. 1A). In addition, we have compared the AMSCs phenotypes before and after hypoxia treatment by flow cytometry. We found that the phenotype of ADSCs was not significantly influenced by 24 hour’s hypoxia treatment (Fig. 1A).

AMSCs have multipotential differentiation ability towards mesenchymal tissues, including bone and adipose tissue. To confirm the differentiation capability of AMSCs, we induced the osteogenic differentiation and adipogenic differentiation of AMSC for 21 days. Alizarin red S staining indicated that aggregation of hydroxyapatite-mineralized matrices in stimulated cells (Fig. 1B). Lipid vacuoles stained by orange-red indicated AMSC could differentiate to adipose cells (Fig. 1B).

### Hypoxia preconditioning up-regulates pro-angiogenic cytokines in rat AMSCs

**Fig 2:**
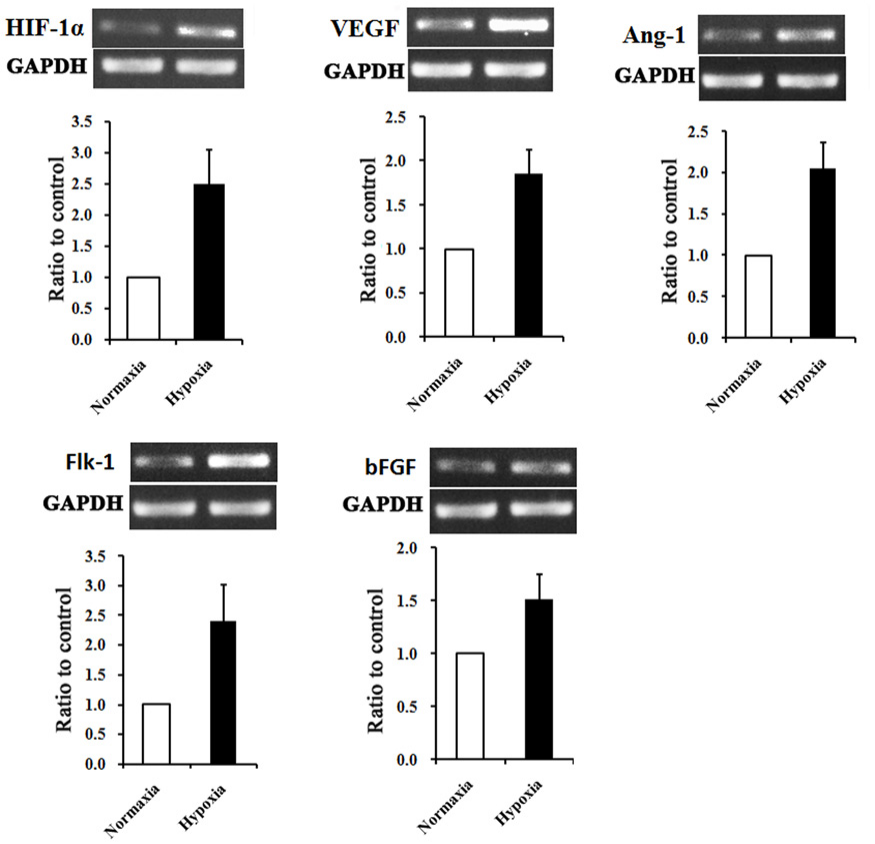
Hypoxia preconditioning upregulates angiogenesis-related factors in AMSCs. RT-PCR demonstrated that several cytokines that were related to neovascularization or vascular protection were significantly unregulated in hypixia-preconditioned AMSCs compared with normoxia ones (*P*<0.01 in each factor).

It has been reported that the living environment of MSCs in vivo is maintained at a low oxygen condition of ~4% [32]. We thus selected the hypoxia treatment of 1% O_2_. As referred to the normoxia in this report, the atmospheric oxygen level of 20% is adopted. Based on previous studies [16,17], it have been confirmed that hypoxia could induce the secretion of cytokines involving in promotion of angiogenesis. We hypothesized that hypoxic incubation could up-regulate the angiogenesis related cytokines. In the study, we found that 24 h exposures of AMSCs to normoxia (20% O_2_) or hypoxia (1%O_2_) did not trigger significant cell death as tested with trypan blue staining (data not shown). RT-PCR analysis showed that AMSCs with normoxia treatment (N-AMSCs) expressed a detectable level of several angiogenic cytokines, including bFGF, VEGF, VEGF receptor Flk-1 and Ang-1 (Fig. 2). Compared with N-AMSCs, the expression of the above angiogenic cytokines was up-regulated in hypoxia preconditioned AMSCs (Fig. 2).

### Hypoxic preconditioning up-regulates neurotrophic factors and regenerative factors in rat AMSCs

**Fig 3:**
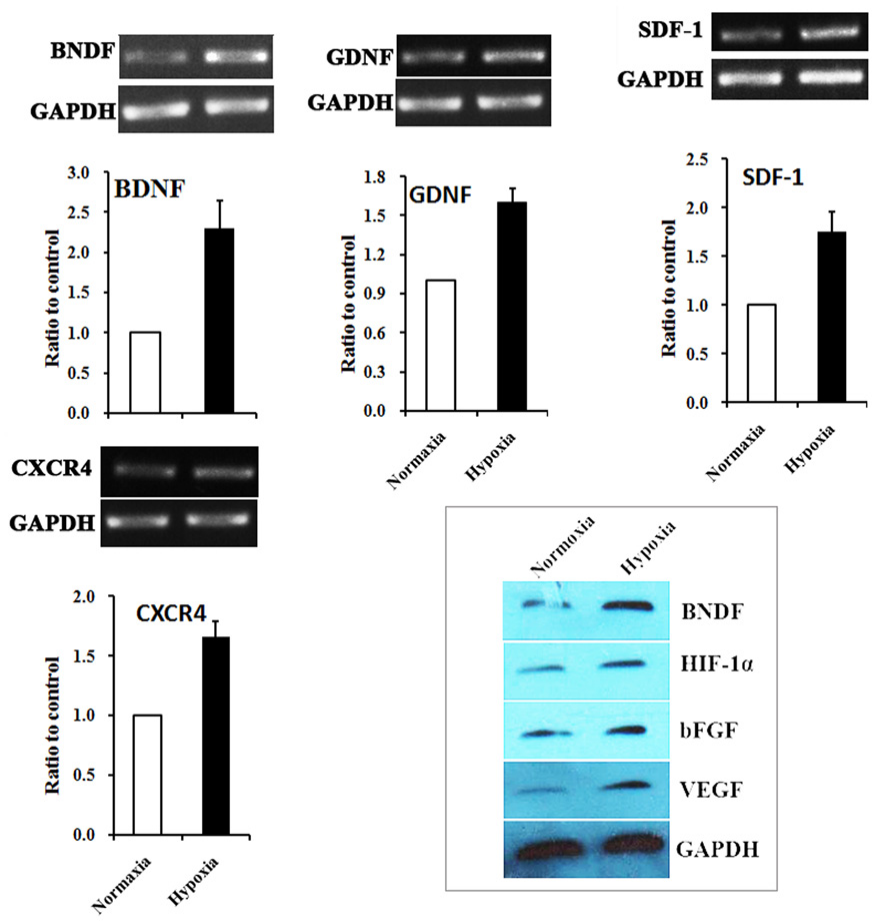
Expression of neurotrophic factors and regenerative factors in AMSCs under normoxia and hypoxia conditions. RT-PCR demonstrated that BNDF and GDNF, important neurotrophic factors, were significantly up-regulated in hypoxia preconditioned AMSCs. Meanwhile, SDF-1 and CXCR4, cytokines closely related with tissue repair, were also significantly up-regulated in AMSCs after hypoxia precondition (*P*<0.01 in each factor).

In addition to angiogenic factors, we also measured the expression of neurotrophic factors and regenerative factors in AMSCs receiving normoxia and hypoxia treatment using RT-PC. The mRNA level of GDNF, which was able to promote the survival and differentiation of neurons [33], was elevated after 24h hypoxia treatment (Fig. 3). We also observed that HP increased the expression of BDNF (Fig. 3), which helps to support the survival of existing neurons [34,35], and promotes the growth and differentiation of new neurons and synapses.The chemokine SDF-1 and its receptor CXCR4 are known to play an important role in mobilizing and directing the migration of neuroblasts [36,37]. The results of RT-PCR showed that hypoxia preconditioning significantly up-regulated the mRNA expression level of SDF-1 and CXCR4 in AMSCs (Fig. 3). The enhanced paracrine effects of AMSCs by hypoxia treatment were further verified by western blotting.

### Survival of transplanted cells in cavernous tissue

**Fig 4:**
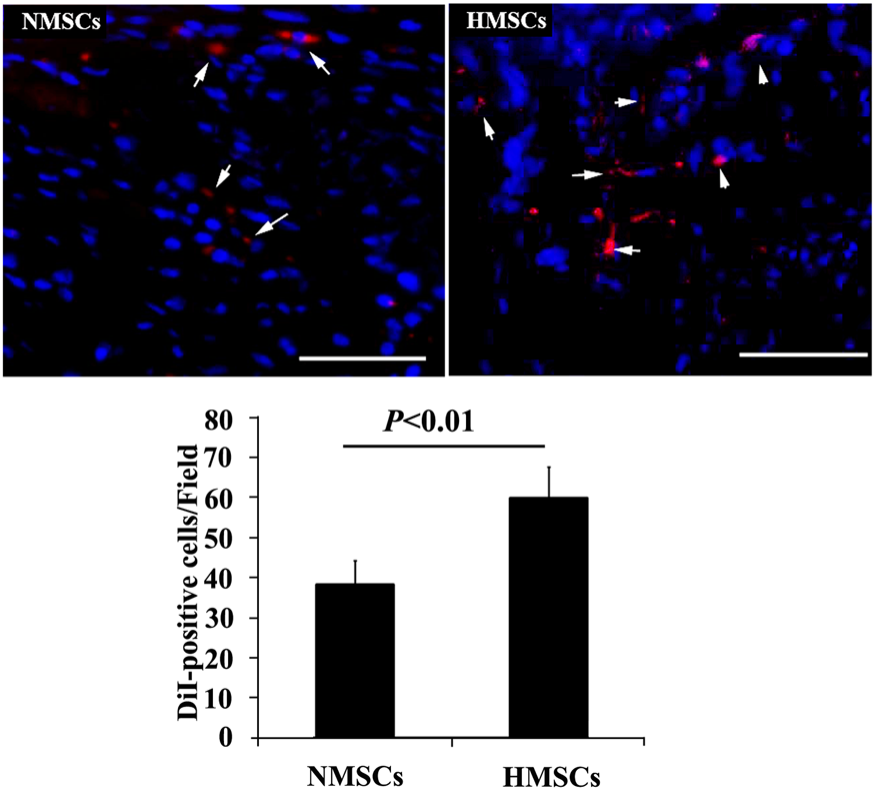
Tracking the engraftment of transplanted cells. Top panel: representative images of DiI-positive AMSCs (red and showed by white arrow) in corpus cavernosum of diabetes induced ED rats receiving intracavernous injection with N-AMSCs (left) and H-AMSCs (right). Bottom panel: The results of quantitative analysis expressed as cell counts summarized per field. More DiI-positive cells were observed in animals receiving HMSCs injection than those receiving NMSCs injection (DAPI staining, Scale bar: 50μm).

Pretransplantation AMSCs were labeled by DiI (Red) for in vivo tracking. 1 week after transplantation, immunofluorescent microscopy was performed on penis sections to analyze the number of survived cells. As shown in Fig. 4, more DiI-positive cells were observed in animals receiving HMSCs injection than those receiving NMSCs injection. This is consistent with the previous reports that hypoxia pretreatment could promote the engraftment and survival of MSCs in host tissue [25,38].

### Transplantation of hypoxic preconditioning AMSCs promoted diabetic erectile function

**Fig 5:**
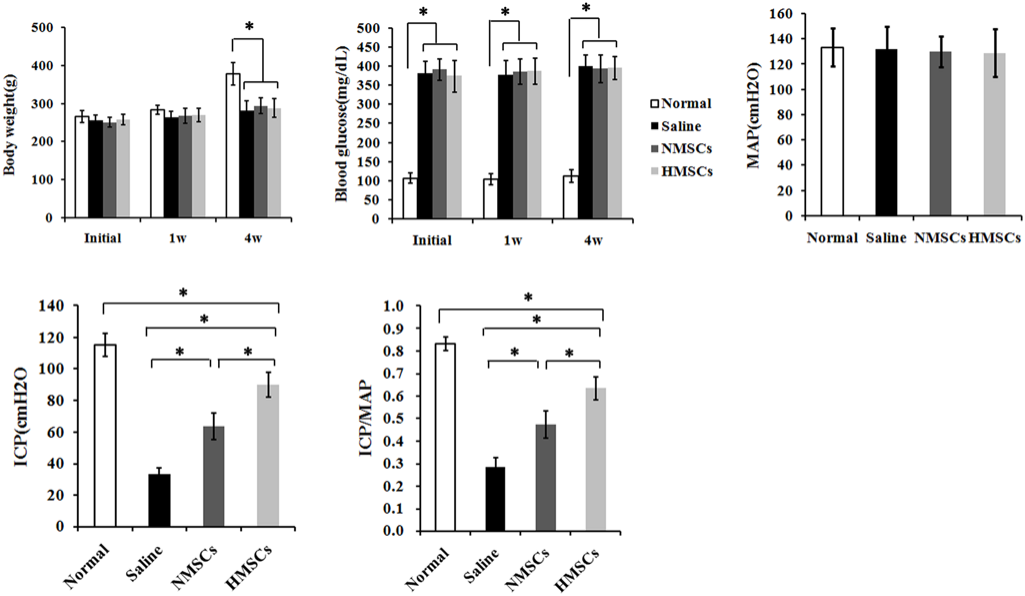
Body weight, blood glucose and erectile function. STZ injection caused a significant body weight loss at the 4st week and continual increase of blood glucose concentration in the diabetic groups compared to age-matched controls. There were no difference among diabetes groups in both body weights and blood glucose level. Erectile function: The ICP value of treatment with N-AMSCs or H-AMSCs is increased and the ratio of total ICP to MAP was also calculated. *P<0.05;

Compared with age-matched nondiabetic controls, a significant decrease of body weights in the diabetic rats was observed 4 weeks after STZ injection (Fig. 5) (*p*<0.05). The injection also triggered a significant increase of blood glucose levels at the beginning, the 1st week and the 4th weak (*p*<0.05). After 4 weeks, both blood glucose concentration and body weights demonstrated no significant difference between MSC treated rats and untreated ones. Measurement of mean arterial pressure (MAP) showed that no significant difference existed between the four groups, indicating that diabetic induction did not affect the MAP of rats.

To compare functional benefits of intracavernous transplantation of H-AMSCs and N-AMSCs in the diabetic induced ED rats, we assessed representative ICP measurements from electrostimulation of the cavernous nerve (CN) (Fig. 5). Rats that received N-AMSCs exhibited a significant trend of improved ICP and peak ICP/MAP ratio in response to CN stimulation compared with saline treated groups (*p*<0.05). Moreover, rats that received H-AMSCs transplantation were observed with significantly better results than those received N-AMSCs transplantation (*p*<0.05).

### Collagen deposition and immune cell infiltration

**Fig 6:**
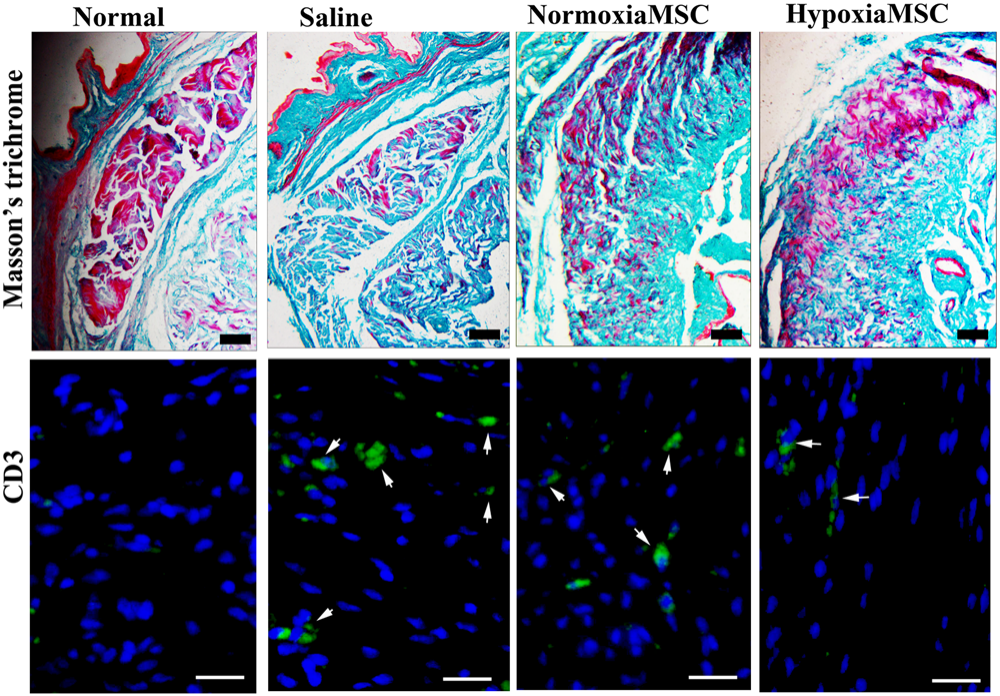
Histological analysis of collagen deposition and histochemical analysis of immune cell infiltration. Top panel: Masson’s trichrome staining to assess the collagen deposition which indicated tissue scarring (Scale bar: 100μm). Bottom panel: representative images of CD3-positive AMSCs (showed by white arrow) in corpus cavernosum of normal control rats (left) and diabetes induced ED rats receiving intracavernous injection with saline (middle left), N-AMSCs (middle right) and H-AMSCs (right). Immunostaining with anti-CD3 antibodies (cell surface markers of T lymphocytes) to assess inflammatory cell recruitment in injured tissue (DAPI staining, Scale bar: 25μm).

Masson’s trichrome staining was performed to assess the collagen deposition which indicated tissue scarring. As shown in Fig. 6, abundant collagen deposition was observed in animals receiving saline injection compared with normal control. While in animals receiving injection of AMSCs, the collagen deposition was apparently reduced compared with those receiving saline injection, and the collagen deposition was further reduced in HAMSCs-treated animals compared with AMSCs-treated ones; To assess inflammatory cell recruitment in injured tissues, immunostaining with anti-CD3 antibodies (cell surface markers of T lymphocytes) was performed and similar results were achieved as that of histology. As shown in Fig. 6, apparent immune cell infiltration was observed in saline-treated animals compared with normal ones, while immune cell infiltration was reduced in AMSCs treated animals compared with saline-treated ones and was further reduced in HAMSCs-treated ones.

### Protective effects of AMSCs on blood vessels in cavernous tissue

**Fig 7:**
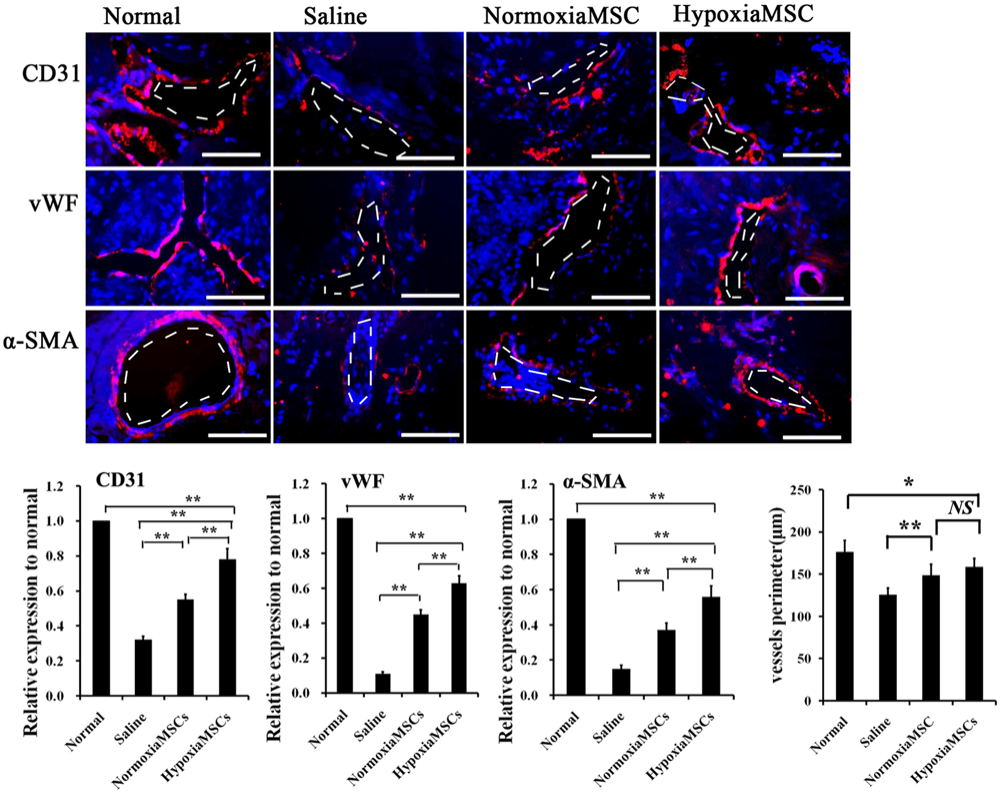
Expression of vascular markers in rats receiving different treatment. Top panel: representative images of CD31, vWF and α-SMA-positive AMSCs (red) in corpus cavernosum of normal control rats (left) and diabetes induced ED rats receiving intracavernous injection with saline (middle left), N-AMSCs (middle right) and H-AMSCs (right). Bottom panel: Ratio of CD31, vWF and α-SMA identified in corpus cavernosum expressed as relative expression to the normal controls. The average vessels perimeter (μm) was calculated by ImagePro software. The saline treated group was significantly but not markedly shorter in length than the AMSCs treated groups, while no significant differentiation was observed between N-AMSCs and H-AMSCs treated group, possibly suggesting limited promotion of structure recovery of vessels. Immunofluorescent staining analysis of cavernous tissue using CD31, vWF and α-SMA antibodies in normal control and saline or N-AMSCs or H-AMSCs treated diabetic rats. Plenty of positive stained cells by CD31, vWF and α-SMA around vessel in the Normal group indicated sound vascular structure. A significant decrease of positive stained cells by CD31, vWF and α-SMA around vessel in the ED induced group (saline group) were observed, suggesting a serious cell damage in vascular structure. When diabetes induced ED rats treated by NMSCs or HMSCs, vascular structure restored to a degree and the HMSCs group had better effects than NMSCs. DAPI staining and vessel: dash line; Scale bar: 50um, *P<0.05, **P<0.01, NS *P*>0.05.

To understand whether AMSC transplantation might have an impact on angiogenesis in the STZ induced diabetic rats, immunofluorescence staining was performed using vascular markers. As showed in Fig. 7, significantly fewer cells expressed endothelial markers (CD31, vWF) in the saline-treated rats than in the normal rats (*p*<0.01). However, N-AMSCs injection partially recovered the endothelial contents compared with the saline injection (*p*<0.01). Moreover, endothelial content in the cavernous tissue after H-AMSCs treatment was further significantly enhanced compared with N-AMSCs injection (*p*<0.01).

When it comes to the vascular smooth muscle, α-SMA expression was analyzed. Among rats that received intracavernous injection of saline, H-AMSCs and N-AMSCs, the cavernous expression of α-SMA was significantly higher in the N-AMSCs treated rats saline treated ones (Fig. 7) (*p*<0.01), while the α-SMA in H-AMSCs treated groups were significantly higher than that in N-AMSCs injected rats (*p*<0.01). The results mentioned above suggested a significantly augmented vascular protection or angiogenesis of H-AMSCs compared with N-AMSCs. In addition, according to results of vessel perimeters, the saline treated group was significantly but not markedly shorter in length than the AMSCs treated groups (*p*<0.01), while no significant differentiation was observed between N-AMSCs and H-AMSCs treated groups (*p*>0.01). This finding may provide a clue of the possible mechanism that AMSCs introduction may mainly promote function but not structure recovery of vessels.

### Expression levels of nNOS

**Fig 8:**
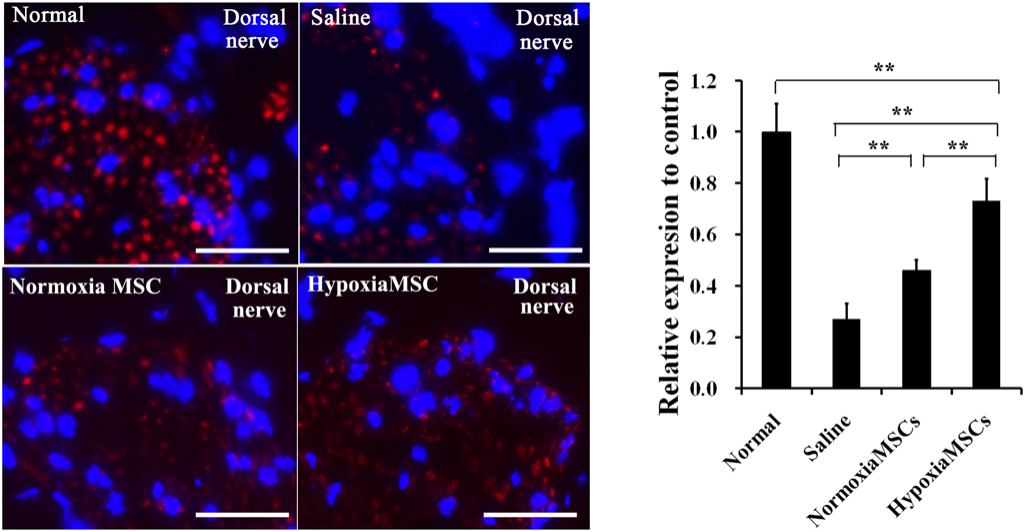
Expression of nNOS in dorsal nerve of rats receiving different treatment. Top panel: representative images of nNOS-positive AMSCs (red) in corpus cavernosum of normal control rats (left) and diabetes induced ED rats receiving intracavernous injection with saline (middle left), N-AMSCs (middle right) and H-AMSCs (right). Bottom panel: Ratio of nNOs identified in corpus cavernosum expressed as relative expression to the normal controls. Immunofluorescent staining analysis of dorsal nerve using nNOS antibodies in normal control and saline or N-AMSCs or H-AMSCs treated diabetic rats. DAPI staining, Scale bar: 50um, **P<0.01.

The secretion of nNOS was regulated by nerve and was crucial for erectile function. To assess the nerve protection of AMSCs after cavernous injection, immunofluorescence staining against nNOS was performed. As shown in Fig. 8, the expression of nNOS in dorsal nerve was significantly lower in saline-treated rats than the N-AMSCs and H-AMSCs treated ones (both p<0.01). When comparison was performed between N-AMSCs and H-AMSCs treated groups, a significant difference was also observed that nNOS expression was higher in H-AMSCs treated group than N-AMSCs treated one (*p*<0.01). Importantly, an almost 70% restoration in the H-AMSCs group was obtained compared with the normal controls.

## Discussion

Nowadays, ED has become a major health problem that severely affects the quality of human life [39]. Advances in the pathophysiologic molecular mechanisms have demonstrated that neurovascular etiology plays an important role during the development of diabetes-associated ED [22]. Though clinically available drugs, such as the phosphodiesterase inhibitor drugs [9], could attenuate the symptom to some extent, the therapeutic effects were not satisfactory. In recent years, stem cell transplantation has emerged as a promising therapy for ED. Mesenchymal stem cells, especially that from adipose tissue (AMSC), have attracted extensive interests due to their unique biology, including abundance of autologous source, ease of isolation as well as being easily expanded [40]. Independent groups have investigated the feasibility of MSCs in treatment of diabetes-related ED and encouraging results have been achieved [21,22], suggesting their potential as a valuable tool for ED repair. However, in general, the investigation about the efficacy of MSCs in therapy of ED was relative preliminary, and the therapeutic potential of the cells is far from being thoroughly explored.

Hypoxia preconditioning has been confirmed to enhance the paracrine effects of MSCs and also been applied for several diseases, such as myocardial infarction [41]. In this study, we utilized the hypoxia preconditioned MSCs for therapy of diabetes-related ED. We found that the secretion of several cytokines that were related to neuro or vascular protection was significantly increased from MSCs after hypoxia precondition, including VEGF, bFGF, BNDF, GDNF, et al. The results were consistent with previous studies [41,42,43]. In addition, flowcytometry analysis demonstrated that the phenotype of ADSCs was not significantly influenced by 24 hour’s hypoxia treatment. We consulted to several documents about the influence of hypoxia on the fate of mesenchymal stem cells. It has been confirmed too by other studies that hypoxia could maintain the undifferentiated state of mesenchymal stem cells within certain time[44], which was consistent with our results. Of course, there are some reports about the induced differentiation of MSCs by hypoxia[45,46], but it needed to cultivate MSCs under hypoxia and differentiation condition for longer time(even more than 2 weeks). After transplantation, hypoxia preconditioned MSCs significantly reduced the vascular and nerve damage as well as significantly improved erectile function compared with normaxia treated MSCs. To the best of our knowledge, this is the first report about the feasibility and efficacy of using hypoxia precondition to augment the therapeutic potential of MSCs in the treatment of diabetes-associated ED. The data of the study provides a promising therapeutic option for stem cell based therapy after ED.

In addition to the paracrine effect, AMSCs were confirmed to possess the potentials to differentiate into various cell types including endothelium, smooth muscle cells, schwann cells, and neurons [47,48]. The engraftment and pluripotency of transplanted stem cells may also contribute to the vascular and nerve regeneration or repair in the corpus cavernosum after ED, which may be another possible mechanism for AMSC based therapy of ED. However, previously, several reports have confirmed that the efficacy of MSCs for tissue repair were mainly through their paracrine effects[49,50,51]. Actually, several studies performed the analysis of the survival of transplanted MSCs in cavernous tissue and consistent results were obtained that rare cells could be detected[22,52]. In the study, we performed experiments to detect the number of transplanted AMSCs in the cavernous tissue by labeling the cells with DiI before transplantation. At one week after transplantation, histological detection showed that more DiI-positive cells could be observed in cavernous tissues receiving H-AMSCs transplantation. This is consistent with the previous studies that hypoxia pretreatment could promote the engraftment and survival of MSCs in host tissue[25,38]. At 4 weeks after transplantation, the in situ differentiation of transplanted cells was also analyzed by staining the tissue sections with vascular markers (CD31 and α-SMA). However, we could hardly observe DiI-positive cells and no DiI-positive cells expressing vascular markers were detected (Data not shown). It could be speculated that hypoxia-pretreatment increased the engraftment of transplanted AMSCs in cavernous tissue in short time but in long time. These results suggested that paracrine effects should be the main mechanism in treatment of ED by AMSC. This may explain why enhanced paracrine effects of AMSCs by hypoxia resulted in the significant improvement in recovery of erectile function, as well as in the vascular and nerve protection in DED rats. To minimize the potential immune rejection in our study, AMSCs were derived from syngeneic rat donors, not human, but we could speculate that hypoxia pretreatment should produce similar effects on therapeutic function of human AMSCs for ED. Actually, several previous studies have investigated the influence of hypoxic pre-conditioning on therapeutic effects of human ADSCs in other animal model[53,54], and it has been confirmed that hypoxic pre-conditioning can improve the therapeutic effect of human ADSCs.

The chemokine receptor 4 (CXCR4) and its ligand, the stromal cell-derived factor-1 (SDF-1), have been believed to be key players in migration and engraftment of endogenous and transplanted stem cells to injured tissues [55]. Furthermore, the two factors were confirmed to play other roles in tissue repair, such as promoting angiogenesis, influencing biological behaviors of MSCs. et al, indicating a significant implication in stem cell therapy. Therefore, several studies have genetically modified MSCs with SDF-1 gene or pretreated MSCs with SDF-1 protein with the purpose of enhancing the therapeutic effects of the cells in tissue repair, and encouraging results were achieved [56,57]. In the study, we also observed the increased expression of SDF-1 and CXCR4 in hypoxia preconditioned MSCs. The phenomenon should also contribute to the recovery of erectile function. However, how much the up-regulated SDF-1 and CXCR4 were related to the improved effectiveness of AMSC in treatment of ED as well as the underlying mechanisms were not clarified in the study. This was a limitation of the study, needing further investigation in the future.

In summary, we demonstrated that several cytokines crucial for vascular and nerve protection were significantly up-regulated in hypoxia preconditioned AMSCs compared with normoxia ones. After in vivo application, the activated paracrine effects contributed to the AMSC-based treatment of ED and finally, we achieved a significant improvement in the therapeutic effects of AMSCs for diabetes associated ED. Overall, the study provide a novel therapeutic option to achieve a better treatment of ED with AMSCs, suggesting a promising perspective in the future clinical practice.
